# Low-dose pioglitazone can ameliorate learning and memory impairment in a mouse model of dementia by increasing LRP1 expression in the hippocampus

**DOI:** 10.1038/s41598-019-40736-x

**Published:** 2019-03-13

**Authors:** Hannah Seok, Minyoung Lee, Eugene Shin, Mi Ra Yun, Yong-ho Lee, Jae Hoon Moon, Eosu Kim, Phil Hyu Lee, Byung-Wan Lee, Eun Seok Kang, Hyun Chul Lee, Bong Soo Cha

**Affiliations:** 1 Department of Internal Medicine, The Catholic University of Korea College of Medicine, Uijeongbu St. Mary’s Hospital, Uijeongbu, Korea; 2 Department of Internal Medicine, Yonsei University College of Medicine, Seoul, Korea; 3 Institute of Endocrine Research, Yonsei University College of Medicine, Seoul, South Korea; 4 Brain Korea 21 Project for Medical Science, Yonsei University College of Medicine, Seoul, Korea; 5 Department of Internal Medicine, Seoul National University Bundang Hospital, Seongnam-si, Korea; 6 Department of Psychiatry, Yonsei University College of Medicine, Seoul, Korea; 7 Department of Neurology, Yonsei University College of Medicine, Seoul, Korea

## Abstract

Amyloid-β (Aβ) accumulation in the brain is a pathological feature of Alzheimer’s disease (AD) and enhancing Aβ clearance is a potential therapeutic strategy. Pioglitazone is a peroxisome proliferator-activated receptor-γ (PPAR-γ) agonist and is widely used to treat type 2 diabetes. We previously reported that low-dose pioglitazone increased the expression of low-density lipoprotein receptor-related protein 1 (LRP1), which upregulates the clearance of Aβ, using human brain microvascular endothelial cells. We investigated whether low-dose pioglitazone can rescue the pathological phenotype and memory impairment in senescence-accelerated mouse prone-8 (SAMP8) mice by increasing LRP1 levels. SAMP8 mice were treated with vehicle or pioglitazone in dosages of 2 or 5 mg/kg/day for 7 weeks. In the water maze test, 2 mg/kg/day of pioglitazone significantly attenuated the increased escape latency in SAMP8 mice (*p* = 0.026), while 5 mg/kg/day of treatment did not. Compared with vehicle treatment, the hippocampi of SAMP8 mice with 2 mg/kg/day of pioglitazone exhibited fewer Aβ deposits and reduced Aβ_1–40_ levels, along with elevated LRP1 expression (*p* = 0.005). Collectively, our results proposed that a new therapeutic application of the PPAR-γ agonist for AD treatment should be considered at a lower dose than the conventional dose used to treat diabetes.

## Introduction

Alzheimer’s disease (AD) is the most common form of senile dementia in the elderly. Accumulation of amyloid-β (Aβ) into the so-called senile plaques, and neurofibrillary tangles are main pathological features of AD^1–3^. Therefore, reducing production of Aβ or increasing the clearance of Aβ from the brain parenchyma could be important treatment strategies for AD^4–6^. A growing body of evidence suggests that the clearance of Aβ is significantly impaired in the majority of patients with AD^5^. Receptor-mediated elimination of Aβ from the brain to the periphery is mainly mediated by low-density lipoprotein receptor-related protein 1 (LRP1)^7^. LRP1, a member of the LDL receptor (LDLR) family, binds a diverse array of extracellular ligands, and is abundantly expressed in various tissues: the liver, brain, and vessels^6,8^. LRP1, expressed in the endothelial cells and pericytes of the blood brain barrier (BBB), is considered to actively eliminate brain-derived Aβ across the BBB^7,9–12^, and dysfunction of LRP1 significantly exacerbates the accumulation of Aβ in the brain^7,13^. Additionally, LRP1 in peripheral tissues such as the liver can also affect Aβ metabolism in the brain by accelerating the uptake of peripherally circulating Aβ^14,15^. Therefore, several pharmaceutical approaches including statin treatment have been made to enhance LRP1-mediated Aβ clearance in *in vitro* and *in vivo* studies^16–19^. Nevertheless, further studies are desired to elucidate the impact of elevated LRP1 on memory improvement in various stages of AD.

Pioglitazone, a peroxisome proliferator-activated receptor gamma (PPAR-γ) agonist, is widely used to treat hyperglycaemia in type 2 diabetes. This PPAR-γ agonist is a potential candidate to treat AD, as it has been shown to improve memory dysfunction and reduce accumulation of Aβ in previous animal studies of AD^20–23^. However, the underlying mechanism remains unknown, and the clinical evidence is controversial^24–26^. Of note, several *in vitro* studies have reported that pioglitazone increases LRP1 expression in multiple types of cells such as adipocytes, hepatocytes, and microvascular endothelial cells^27–29^. Interestingly, Moon *et al*. reported that low-dose PPAR-γ agonist treatment, but not the conventional doses, exhibits an Aβ-clearing effect by increasing LRP1 in human brain microvascular endothelial cells (HBMECs)^29^. Another PPAR-γ agonist, rosiglitazone, upregulates mRNA and protein levels of LRP1 in addition to LRP1 promoter activity, and increases Aβ uptake via LRP1 in HBMECs^29^. This increase in LRP1 expression and Aβ uptake was observed for concentrations of ≤10 nM of PPAR-γ agonist, but not for concentrations ≥20 nM. Considering that a 7-day rosiglitazone oral treatment results in concentrations of 260–450 nM in the human brain^30^, this unusual dose-response *in vitro* study suggests that a new therapeutic application of PPAR-γ agonist for AD should be considered at a lower dose than the conventional dose used to treat diabetes. Therefore, it is of great interest to examine whether low-dose pioglitazone can reduce Aβ plaque deposition and ameliorate memory impairment in mouse model of AD by increasing LRP1 expression.

In this study, we investigated whether pioglitazone could upregulate LRP1 expression, accompanied by reduction of Aβ plaque deposition in a mouse model of sporadic AD, senescence-accelerated mouse prone-8 (SAMP8). The administered dosages of pioglitazone were 2 or 5 mg/kg/day, comparable with 10 and 26 mg/day by human equivalent dose calculation, respectively^31,32^. Our finding suggests a theoretical basis for the use of pioglitazone in treating AD, by demonstrating the efficacy of low-dose pioglitazone in the improvement of memory impairment and Aβ pathology-related LRP1 expression in a mouse model of AD.

## Results

### Low-dose pioglitazone improves spatial learning and memory deficits in aged SAMP8 mice

**Figure 1:**
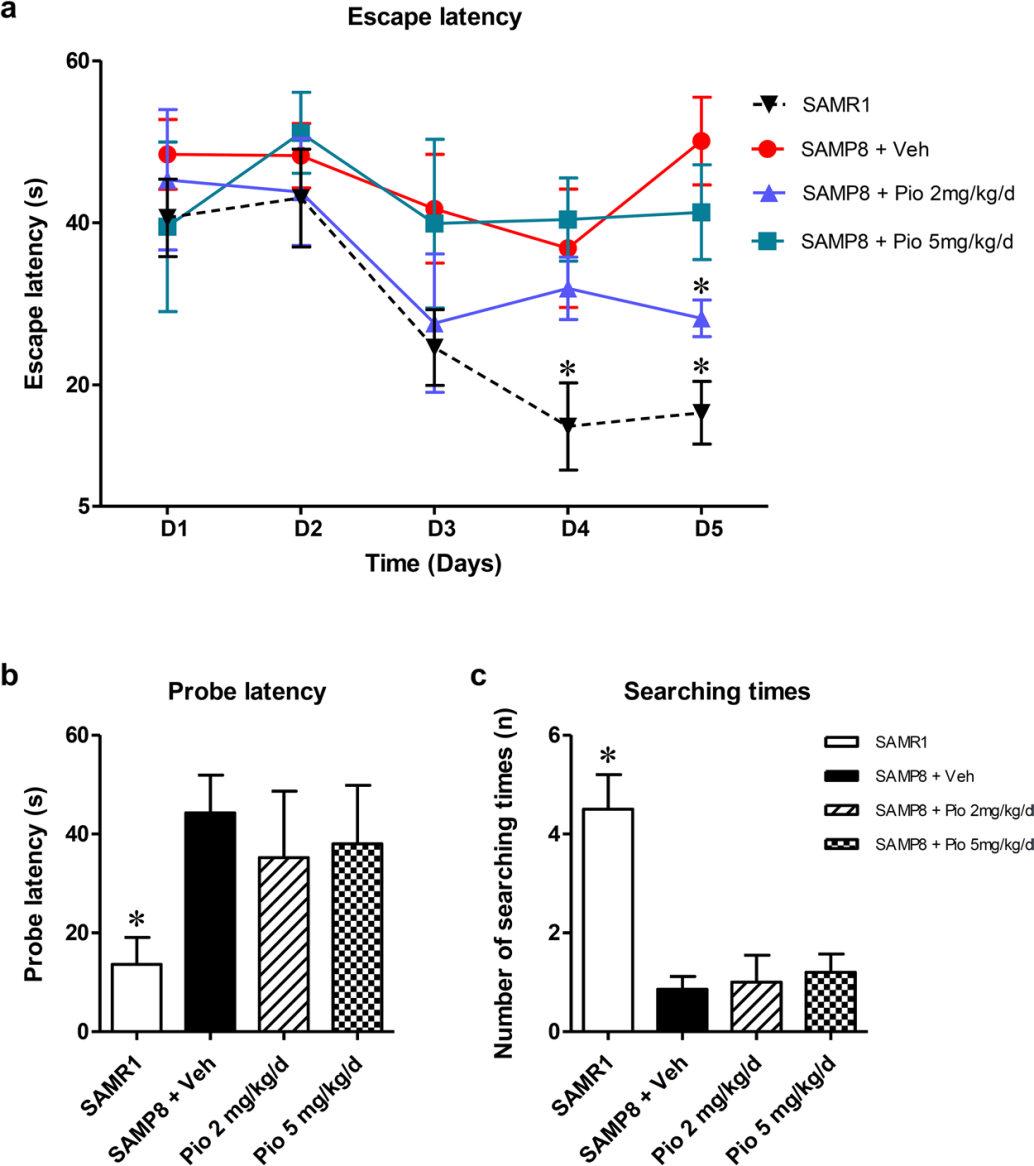
Pioglitazone improves spatial learning and memory in SAMP8 mice. (**a**) Effect of pioglitazone on escape latency in training trial sessions. On the 5th day of the training trial, SAMR1 mice and SAMP8 mice treated with 2 mg/kg/day of pioglitazone exhibited significantly decreased escape latency compared with vehicle-treated SAMP8 mice. In the probe trial session, (**b**) probe latency and (**c**) searching times were not significantly improved by treatment with 2 or 5 mg/kg/day of pioglitazone compared with vehicle treatment in SAMP8 mice. The data are expressed as the mean ± standard error of the mean (n = 7 for vehicle; n = 5 for each dosage of pioglitazone treatment group; and n = 10 for SAMR1 mice). *p < 0.05 compared with  vehicle-treated SAMP8 mice.

In training trials, 11-month-old SAMP8 mice exhibited spatial learning and memory impairment compared with SAMR1 mice in the water maze test (Fig. 1a). The escape latency (time taken to find the hidden platform) tended to improve in SAMR1 mice, but not in SAMP8 mice in repeated trials. Notably, 2 mg/kg/day of pioglitazone significantly attenuated the escape latency in SMAP8 mice on the 5th day (*p* = 0.026). The escape latency was also decreased by 5 mg/kg/day of pioglitazone compared with vehicle treatment in SAMP8 mice on the 5th day, but the difference was not statistically significant (*p* = 0.274).

In the probe trials, decreased probe latencies and increased searching times were achieved by 2 or 5 mg/kg/day of pioglitazone treatment compared with vehicle treatment in SAMP8 mice. However, these improvements were not statistically significant (Fig. 1b,c).

### Low-dose pioglitazone decreases Aβ plaque depositions and Aβ_1–40_ levels

**Figure 2:**
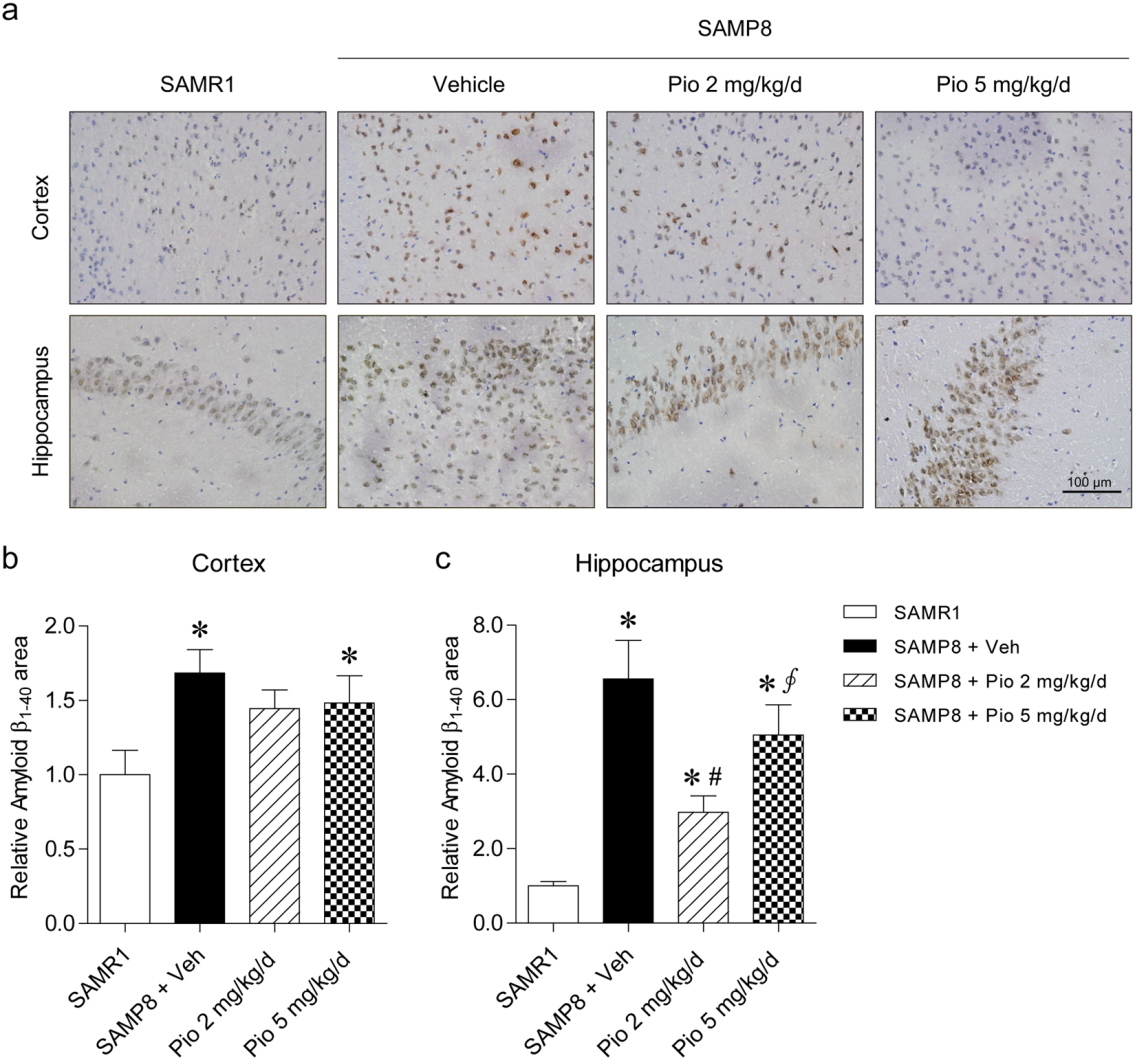
Immunohistochemistry for Aβ_1–40_ deposits in the cortices and hippocampi of SAMR1 and SAMP8 mice. (**a**) Representative immunohistochemical staining images are shown. The relative area covered by Aβ_1–40_ plaques in (**b**) cortices and (**c**) hippocampi of SAMR1 and SAMP8 mice was analyzed. The Aβ_1–40_ deposits were increased in the cortices and hippocampi of SAMP8 mice compared with SAMR1 mice. The Aβ_1–40_ deposits were reduced in the hippocampi of SAMP8 mice treated with 2 mg/kg/day of pioglitazone compared with vehicle-treated SAMP8 mice, but the difference was not significant in the cortices. Values are the mean ± standard error of the mean (n = 8 per each group). *p < 0.05 compared with SAMR1 mice. ^#^*p* < 0.05 compared with vehicle-treated SAMP8 mice. ∮*p* < 0.05 compared with SAMP8 mice treated with 2 mg/kg/day of pioglitazone. The scale bar represents 100 μm.

Immunohistochemical staining for Aβ_1–40_ revealed significantly increased Aβ deposits in the cortices and hippocampi of vehicle-treated SAMP8 mice compared with SAMR1 mice (Fig. 2). The Aβ_1–40_ deposits were markedly reduced in the hippocampi of SAMP8 mice treated with 2 mg/kg/day of pioglitazone compared with vehicle-treated SAMP8 mice (*p* = 0.013; Fig. 2c), but the difference was not apparent in the cortices (Fig. 2b). Compared with vehicle treatment, 5 mg/kg/day of pioglitazone treatment did not reduce Aβ_1–40_ deposits, neither in the hippocampi nor in the cortices of SAMP8 mice (Fig. 2b,c). Similarly, immunohistochemical staining for Aβ_1–42_ exhibited that vehicle-treated SAMP8 mice had a significantly increased Aβ_1–42_ deposits in the cortices and hippocampi of vehicle-treated SAMP8 mice compared with SAMR1 mice, and 2 mg/kg/day of pioglitazone treatment significantly diminished Aβ_1–42_ levels compared with vehicle treatment in the hippocampi of SAMP8 mice (*p* = 0.005; Supplementary Fig. S1).

**Figure 3:**
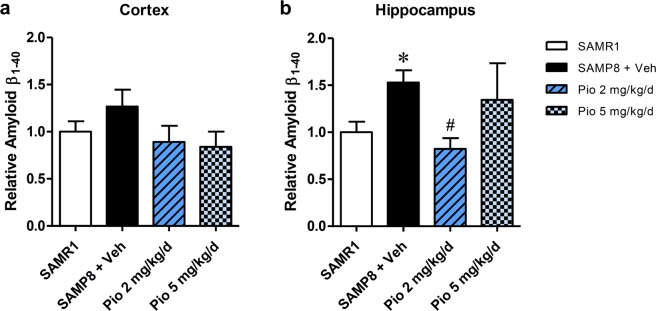
Effect of pioglitazone on the levels of Aβ_1–40_ in brain regions of SAMR1 and SAMP8 mice. (**a**) Cortical and (**b**) hippocampal relative levels of Aβ_1–40_ were determined by ELISA. SAMR1 mice were used as the reference group. Values are the mean ± standard error of the mean (n = 4–5 for cortical Aβ_1–40_ levels per group; and n = 5–8 for hippocampal Aβ_1–40_ levels per group). *p < 0.05 compared with SAMR1 mice. ^#^*p* < 0.05 compared with vehicle-treated SAMP8 mice.

Cortical and hippocampal levels of Aβ_1–40_ were also determined by enzyme-linked immunosorbent assay (ELISA). SAMP8 mice had higher soluble Aβ_1–40_ levels than SAMR1 mice in both the cortical and hippocampal areas, but the difference was only statistically significant in the hippocampi (*p* = 0.005; Fig. 3a,b). Compared with vehicle treatment, treatment of SAMP8 mice with pioglitazone (2 or 5 mg/kg/day) in SAMP8 mice did not significantly reduce Aβ_1–40_ levels in cortices (Fig. 3a). SAMP8 mice treated with 2 mg/kg/day of pioglitazone exhibited a significantly lower level of Aβ_1–40_ in the hippocampal area, which is involved in spatial learning and memory, than the vehicle-treated SAMP8 mice (*p* = 0.004; Fig. 3b). A higher dosage of pioglitazone (5 mg/kg/day) did not significantly reduce Aβ_1–40_ levels in the hippocampi of SAMP8 mice, compared with vehicle treatment (*p* = 0.329; Fig. 3b).

### Low-dose pioglitazone increases LRP1 expression in the microvessels of the hippocampal area

**Figure 4:**
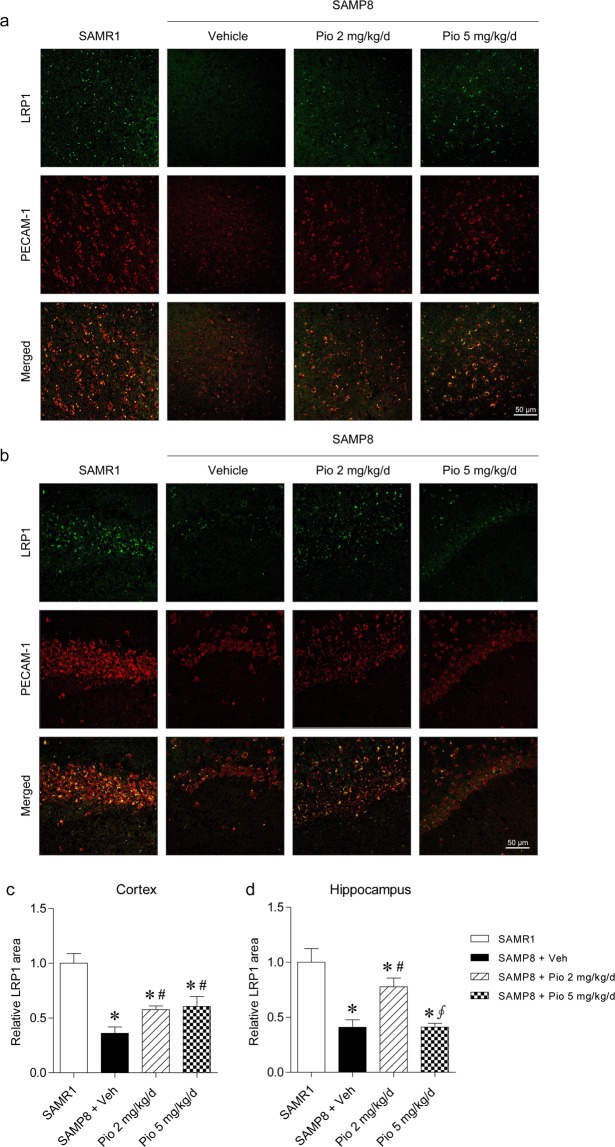
Immunofluorescence staining for LRP1 and PECAM-1 in (**a**) the cortex and (**b**) the hippocampus. LRP1 (green) expression was merged with that of PECAM-1 (red). The relative LRP1 positive area was quantified in (**c**) cortices and (**d**) hippocampi of SAMR1 and SAMP8 mice. Compared with SAMR1 mice, SAMP8 mice exhibited decreased LRP1 expression in the cortex and hippocampus. Decreased LRP1 expression in the hippocampi of vehicle-treated SAMP8 mice was significantly improved by 2 mg/kg/day of pioglitazone treatment. Values are the mean ± standard error of the mean (n = 8 per group). *p < 0.05 compared with SAMR1 mice. ^#^*p* < 0.05 compared with vehicle-treated SAMP8 mice. ^∮^*p* < 0.05 compared to SAMP8 mice treated with 2 mg/kg/day of pioglitazone. The scale bar represents 50 μm.

LRP1 expression in the cortex and hippocampus was evaluated in conjunction with platelet endothelial cell adhesion molecule-1 (PECAM-1), an endothelial marker, because LRP1 in the BBB has been demonstrated to be an important exporter of Aβ (Fig. 4) Immunofluorescence double staining for LRP1 and PECAM-1 revealed that LRP1 was distributed along the blood vessels in the brain (Fig. 4a,b). Quantification of the LRP1 positive area revealed that vehicle-treated SAMP8 mice exhibited significantly lowered LRP1 expression than SAMR1 mice in both cortical and hippocampal areas (*p* = 0.001 for both cortical and hippocampal LRP1 levels; Fig. 4c,d). LRP1 expression in the hippocampus, which modulates brain Aβ clearance, was inversely correlated with hippocampal Aβ levels. LRP1 expression was 1.9-fold increased by 2 mg/kg/day of pioglitazone treatment compared with vehicle treatment in the hippocampi of SAMP8 mice (*p* = 0.005), whereas 5 mg/kg/day of pioglitazone treatment showed no significant change on hippocampal LRP1 expression (*p* = 0.753; Fig. 4d). In the cortical area, the difference in LRP1 expression was relatively not considerable between vehicle and pioglitazone treatment at either dosages (Fig. 4c).

LRP1 levels were also analyzed in the cortex and hippocampus by western blot (Supplementary Fig. S2). In the hippocampal area, the protein levels of LRP1 were decreased in vehicle-treated SAMP8 mice compared with SAMR1 mice (*p* = 0.034). SAMP8 mice treated with 2 mg/kg/day of pioglitazone which exhibited lower hippocampal Aβ_1–40_ levels, had higher levels of hippocampal LRP1 than vehicle-treated SAMP8 mice (*p* = 0.050). In the cortical area, there were no statistically significant differences in the protein levels of LRP1 between the groups (*p* = 0.776).

## Discussion

Compared with SAMR1 mice, SAMP8 mice exhibit significant learning and memory dysfunction between 8 and 12 months of age due to impairment in neuroprotection, signal transduction, and enhanced Aβ production, all of which are processes involved in learning and memory^33–36^. In this study, we demonstrated that escape latency during the water maze test was significantly improved by treatment with 2 mg/kg/day of pioglitazone in the SAMP8 mice. Treatment with 2 mg/kg/day of pioglitazone reduced Aβ deposition in SAMP8 mice, and significantly increased the hippocampal expression of LRP1, which plays a key role in the clearance of Aβ in the brain^9–11^.

AD is the most common type of dementia and is a debilitating neurodegenerative disease^37,38^. Nevertheless, no effective treatment has yet been developed and AD remains incurable^38^. Recently, ample evidence has suggested that a PPAR-γ agonist could be a potential therapeutic candidate for the treatment of AD^39–41^. PPARs are nuclear receptors that act as ligand-activated transcriptional regulators of genes affecting lipid metabolism^20,42^. PPAR-γ is the most studied isoform of the PPAR family, and controls adipocyte differentiation along with fatty acid and glucose metabolism^39,43^. Regarding the treatment of type 2 diabetes, PPAR-γ agonists decrease plasma fatty acid and hyperglycaemia by improving insulin sensitivity^39^. Pioglitazone is one such PPAR-γ agonist, and has been clinically used as an anti-diabetic drug since the 1990s^42^. In a prospective cohort study of 145,928 subjects with type 2 diabetes aged ≥60 years, long-term use of pioglitazone was significantly associated with a lower dementia incidence with a relative risk of 0.53^40^. In another prospective randomized trial, 6 months of pioglitazone treatment significantly decreased AD assessment scale and Wechsler memory scale scores in contrast to the control group who did not exhibit any improvement^41^. Several animal studies have also reported the effect of PPAR-γ agonists on memory dysfunction in AD mouse models. A previous study reported that pioglitazone treatment (9 or 18 mg/kg/day for 9 days) improved spatial memory in a mouse model of scopolamine-induced memory impairment^44^. It has also been reported that rosiglitazone treatment (5 mg/kg/day for 4 weeks) in amyloid precursor protein (APP) mice improved recognition memory but not spatial memory^23^. Another study reported that chronic treatment with rosiglitazone (30 mg/kg of food for 7 months) rescued the impaired spatial memory^45^.

Although still largely unknown, PPAR-γ agonists are expected to ameliorate memory impairment in AD by several mechanisms. Animal studies have demonstrated that PPAR-γ agonists inhibit neuroinflammation, improve mitochondrial function and synapse plasticity, and alleviate tau hyperphosphorylation^26,39,46^. Regarding Aβ accumulation in AD, previous animal studies demonstrated that Aβ production is not affected by treatment with a PPAR-γ agonist in the amyloidogenic mouse model^23,47^. Instead, accumulated evidence suggests that PPAR-γ agonists reduce Aβ plaques by enhancing Aβ clearance^47–49^. In the amyloid precursor protein/presenilin 1 (APP/PS1) mouse model, treatment with pioglitazone facilitated the clearance of Aβ via overexpression of apolipoprotein E (ApoE)^47^. Additionally, in another study using the APP/PS1 mouse model, the PPAR-γ agonist enhanced microglial phagocytosis of Aβ via upregulation of scavenger receptor CD 36 expression^49^.

However, to the best of our knowledge, none of the previous reports attempted to investigate the mechanism underlying PPAR-γ agonist-mediated Aβ clearance in association with LRP1 using an AD mouse model. LRP1 has received considerable attention as a therapeutic target of AD, as preclinical studies indicated that LRP1 largely contributes to the pathogenesis of AD by modulating Aβ clearance as well as via an Aβ-independent mechanism^6,7^. Although previous reports have evaluated the effect of pioglitazone on LRP1 expression in the brain using animal models, those mouse models had metabolic features of diabetes; thus, it was difficult to exclude the anti-diabetic effect of pioglitazone from the results^50,51^. Our study is the first to investigate whether LRP1 level is associated with improved memory impairment using the PPAR-γ agonist, pioglitazone, in the SAMP8 mouse model. In our study, a reduction of Aβ burden was observed after pioglitazone treatment, and immunofluorescence staining of LRP1 merged with the endothelial marker, PECAM-1, revealed increased LRP1 expression in pioglitazone-treated SAMP8 mice. SAMP8 mice treated with 2 mg/kg/day of pioglitazone exhibited the greatest reduction in Aβ levels along with the greatest increase in LRP1 levels in the hippocampus, suggesting that elevated LRP1 was closely related to reduction of Aβ. Furthermore, improvements in the water maze test were only observed in the group treated with 2 mg/kg/day of pioglitazone. This result also supported the notion that increased LRP1 with reduction of Aβ in the hippocampus could be responsible for the learning and memory improvement promoted by pioglitazone treatment.

Despite considerable experimental evidence from *in vitro* and *in vivo* models, the efficacy of PPAR-γ agonists in AD treatment remains controversial^24–26,37,52^. In a meta-analysis encompassing nine clinical studies of 1,314 patients and 1,311 control subjects, statistical evidence was insufficient to support the effect of a PPAR-γ agonist on memory improvement in patients with AD and mild-to-moderate AD^37^. In a previous 18-month randomized controlled trial of pioglitazone, no treatment effects were observed on the efficacy outcomes (measures of cognition, activities of daily living, neuropsychiatric symptoms, and global function)^24^. Of note, in the majority of previous clinical studies, the PPAR-γ agonist was applied at conventional doses used to treat type 2 diabetes or even higher^24,25,37,52,53^. A previous animal study using Wistar rats reported that the functional connectivity with the CA1 region of the hippocampus, a region responsible for memory that is impaired in early AD, was significantly increased by pioglitazone treatment at 0.08 mg/kg/day^54^. The smallest increase in functional connectivity with the CA1 region was observed at the highest pioglitazone dose^54^, suggesting a better therapeutic potential of lower dose pioglitazone compared with higher doses. Furthermore, recent in *vitro* studies have shown that rosiglitazone increases LRP1 expression and Aβ uptake in microvascular endothelial cells at concentrations approximately 10- to 20-fold lower than clinically-used doses^29^. Similar to the previous results of in *vitro* studies, we administered pioglitazone at doses approximately 10- to 20-fold lower than the generally used doses of 20–40 mg/kg/day in other previous animal studies^20,26,55^. Our study proved that 2 mg/kg/day of pioglitazone had an effect on Aβ accumulation and LRP1 expression, with significant learning and memory improvement in an AD mouse model. This unusual dose administration awards our data a novelty status. Current findings support that low-dose pioglitazone should be considered in the attempt to find a new therapeutic application of PPAR-γ agonists in AD. PPAR-γ agonists have pleiotropic physiological functions, and are expected to play a beneficial role in treating AD by regulating multiple aspects in the pathogenesis of AD^26^. Conventional and higher doses of pioglitazone may have beneficial effects on AD. The possibility of pioglitazone’s beneficial effects on AD in various dosages and via mechanisms other than the one involving LRP1 still exists. However, we focused on that low-dose pioglitazone could positively impact on the LRP1 molecular pathway during the development of AD in this study. Since previous clinical studies of pioglitazone at conventional doses yielded conflicting results, future studies should consider a lower dose targeting an increase of LRP1 levels.

This study is not without limitations. First, unfortunately, the LRP1 levels in the microvasculature and parenchyma tissues were not quantified separately in this study. A recent study reported that LRP1 expression is predominant in neurons but is down-regulated in the brain microvasculature in AD^56^. These results also suggested that the role of LRP1 expression might differ between the microvasculature and the parenchyma. Thus, follow-up studies should be performed to clarify the role of LRP1 in AD in addition to the effects on LRP1 expression in microvessels and other areas of the brain. Second, a motor activity test such as the rotarod test was not performed to confirm any motoric disabilities that could have affected the swimming, although learning in the water maze test basically relies on motor performance^57,58^. Third, we could not demonstrate any improvement by pioglitazone treatment in the probe trials to assess retention memory. This unimproved retention memory could be caused by the ceiling effect of aging, and further study using mice of different ages needs to be conducted.

Collectively, our data demonstrated that low-dose pioglitazone ameliorates AD pathology and restores spatial learning and memory impairment in SAMP8 mice. As we hypothesized, pioglitazone possibly facilitated the clearance of Aβ, via activation of LRP1 in the hippocampus. Considering that no effective drug has been developed to treat AD, our study proposed the novel therapeutic potential of PPAR-γ agonist for AD treatment at a lower dose than the conventional clinical dose to treat diabetes.

## Methods

### Animals and drug treatments

SAMP8 mice representing the behavioural and pathological features of late-onset and age-related sporadic AD were used in this study^59,60^. Considering the life span and age-related memory impairment of SAMP8 mice^34,60–63^, 9-month-old male SAMP8 mice were purchased from Japan SLC, Inc., Shizuoka, Japan. Animals were housed individually with food and water *ad libitum* and maintained under controlled conditions (12 h light/dark cycle; 25 ± 2 °C). Age-matched senescence-accelerated-resistant mice 1 (SAMR1) were used as a normal aging control and treated with vehicle by oral gavage for 7 weeks (n = 10). SAMP8 mice were treated with vehicle or pioglitazone hydrochloride (AD-4833, Takeda Pharmaceuticals, Kanagawa, Japan) as a suspension in sterile water by oral gavage at dosages of 2 or 5 mg/kg/day for 7 weeks (n = 7 for vehicle; and n = 5 for each dosages of pioglitazone). In all cases, animals were euthanized 24 h after the final administration. The brains were then removed and immediately frozen on dry ice before dissection. All animal experiments in this study were approved by the Institutional Animal Care and Use Committee of Yonsei University Health System (YUHS-IACUC). The YUHS-IACUC has regulations, notifications, and guidelines that are in accordance with the Animal Protection Act (2008), the Laboratory Animal Act (2008), and the Eighth Edition of the Guide for the Care and Use of Laboratory Animals of NRC (2011). Thus, all animal experiments in this study have been performed in accordance with the guidelines and regulations of the YUHS-IACUC as described above.

### Cortex and hippocampus slice preparation and immunohistochemistry

Brain tissues from mice were fixed in 10% formaldehyde and embedded in paraffin. The paraffin sections were cut and deparaffinized using xylene and ethanol. After inactivation of endogenous peroxidase with 3% H_2_O_2_ for 10 min at 22 ± 2 °C, samples were blocked with 5% bovine serum albumin (BSA) for 1 h. The tissues were then incubated overnight with a mouse anti-amyloid β_1–40_ antibody (1:100, AnaSpec Inc, Fremont, CA, USA) or a mouse anti-amyloid β_1–42_ antibody (1:100, AnaSpec Inc, Fremont, CA, USA) at 4 °C. After washing, tissues were incubated with an appropriate secondary antibody (horseradish peroxidase-labelled coat anti-mouse IgG, 1:1000; ZSJQ) for 1 h at room temperature. Staining for mouse primary antibodies was performed using an EnVision + System-HRP Labelled Polymer Anti-Mouse kit (K4001, Dako, Glostrup, Denmark). Coverslips were washed and incubated with 3,3′-diaminobenzidine (DAB; K3468, Dako) for 5 min and counterstained with haematoxylin prior to examination under a light microscope (Olympus BX40, Olympus Optical Co. Ltd., Tokyo, Japan). Images of immunohistochemically stained sections were captured using an Olympus DP71 microscope digital camera and the relative levels Aβ_1–40_ and Aβ_1–42_ were quantified using Image J software. After adjusting for the threshold within the section image, the percent surface area above the threshold was then measured to determine the total area of the Aβ plaques and the percentage of the total brain area occupied by the Aβ plaques. Data pooled from 4 slide sections at ×400 magnification per each mouse were used for statistical analysis.

### Immunofluorescence

Brain tissues from mice were fixed in 10% formaldehyde and embedded in paraffin. The paraffin sections were cut and deparaffinised using xylene and ethanol. Deparaffinised sections were underwent antigen retrieval in Proteinase K (Proteolytic enzyme solution diluted in 0.05 M Tris-HCl, 15 mM sodium azide, pH 7.5) at room temperature for 10 min. The brain sections were then washed and blocked for 1 h at room temperature with 5% BSA. The slides were then incubated with a rabbit anti-LRP1 antibody (ab199567; 1:200, Abcam^®^, Cambridge, MA, USA) and a mouse anti-PECAM-1 antibody (ab24590; 1:200, Abcam^®^, Cambridge, MA, USA) overnight at 4 °C. After three 5-min washes in Tris-buffered saline (TBS), the slides were incubated with an Alexa 488-conjugated chicken anti-rabbit IgG antibody (A21441; 1:500, Invitrogen, Carlsbad, CA, USA) and an Alexa 647-conjugated goat anti-mouse IgG antibody (ab150115; 1:500, Abcam^®^, Cambridge, MA, USA) in blocking buffer for 30 min at 22 ± 2 °C. The sections were then washed and mounted on coverslip with fluorescent mounting media (Dako). Fluorescence images were examined using a Zeiss Laser Scanning Confocal Microscope (LSM 700, Carl Zeiss, Oberkochen, Germany) and the Zen 2012 software (Zeiss) was used for image processing. All images were acquired using identical acquisition parameters as 24-bit, 1024 × 1024 arrays. The relative levels of immunofluorescence staining for LRP1 were quantified using Image J software. After adjusting for the threshold to isolate specific fluorescence within the section image, the percent surface area above the threshold was then measured to determine the relative LRP1 positive area. Data pooled from 4 slide sections at × 400 magnification per each mouse were used for the statistical analysis.

### ELISA measurement of Aβ_1–40_

Soluble Aβ_1–40_ levels in the cortex and hippocampus were measured with a sensitive sandwich ELISA kit (Catalogue # KMB3481, Thermo Scientific, Rockford, IL, USA). Briefly, the tissue was weighed and homogenized in 100 μl of phosphate-buffered saline (PBS) followed by centrifugation at 16,000× g for 10 min at 4 °C. The supernatant was diluted with standard diluent buffer supplemented with Halt^®^ protease inhibitor cocktail (Thermo Scientific, Rockford, IL, USA). A total of 600 pg/ml was loaded onto ELISA plates in duplicate, and the manufacturer’s instructions were followed.

### Western blot analysis

Proteins were extracted from the homogenized frozen brain tissue using TPER^®^ (Thermo Scientific) mixed with Halt^®^ protease inhibitor cocktail (Thermo Scientific). Protein concentrations were determined using a BCA Protein Assay kit (Thermo Scientific) and appropriate amounts of protein were mixed with sodium dodecyl sulphate (SDS) sample buffer [62.5 mM Tris-HCl pH 6.8, 25% (v/v) glycerol, 2% SDS (w/v), 0.01% (w/v) bromophenol blue, 5% (v/v) β-mercaptoethanol]. Lysates (20 μg per sample) were separated by 10% (w/v) SDS-polyacrylamide gel electrophoresis (PAGE), and the resolved proteins were transferred onto nitrocellulose membranes (GE Healthcare Life Sciences, Pittsburgh, PA, USA). Blots were blocked with 5% w/v non-fat milk in TBST (TBS containing 0.1% v/v Tween 20) for 1 h and incubated with a rabbit anti-LRP1 antibody (1:50,000, Abcam^®^, Cambridge, MA, USA) overnight at 4 °C. After incubation with horseradish peroxidase-conjugated goat anti-rabbit IgG (1:10,000, Thermo Scientific) for 1 h at 22 ± 2 °C, signals were detected using SuperSignal^®^ West Pico Chemiluminescent Substrate reagent (Thermo Scientific). Images were captured and analyzed with a LAS-4000 luminescent image analyzer (Fujifilm Life Science, Stamford, CT, USA)

### Morris water maze

Animals underwent spatial reference learning using the Morris water maze test after 7 weeks of vehicle or pioglitazone treatment with minor modifications from the previously reported methods^44,64,65^. The Morris water maze consists of a large circular pool (90 cm in diameter, 45 cm in height), filled to a depth of 30 cm with water at 20 ± 3 °C. The water was made opaque with a non-toxic black coloured dye. The pool was divided arbitrarily into four equal quadrants. A submerged platform was centred in one of the target quadrants of the pool and submerged 1 cm below the water surface. The position of the platform was unaltered throughout the training trial sessions. Basic training was composed of a hidden-platform acquisition training session and a probe trial session.

The training trials were performed twice daily for 5 consecutive days. At the start of each training trial, mouse was placed on the platform for 15 s and then randomly placed in the water pool. Each trial was terminated when the mouse reached the platform or after 60 s, whichever occurred first. The time taken to find the hidden platform (escape latency) was recorded in each trial. After completing the final training trial session, the mice were subjected to a probe-trial session in which the platform was removed from the pool. Probe-trials were performed with a cut-off time of 60 s and determined whether mice could find the previous platform site. Probe latency (the initial time that the mice crossed the former platform) and searching times (the number of times that the mice crossed the former platform) were monitored by a camera mounted in the ceiling directly above the pool, and all trials were recorded using a water maze program.

### Statistical analysis

Data are expressed as the mean ± standard error of the mean. For animal experiments, data collection and analysis were performed blinded to the condition of the experiments. Statistical significance was confirmed using the Mann Whitney U-test when comparing two groups or the Kruskal-Wallis test when comparing more groups. *P*-values of < 0.05 were considered as statistically significant. All statistical analyses were conducted using SPSS software, version 23.0, for Windows (IBM Corp., Armonk, NY, USA).

## Supplementary information

Supplementary Information

## Data Availability

All data generated or analyzed during this study are included in this published article.
