# Supplementary material for: Low-dose pioglitazone can ameliorate learning and memory impairment in a mouse model of dementia by increasing LRP1 expression in the hippocampus
Source: Sci Rep. 2019 Mar 13;9:4414. doi: 10.1038/s41598-019-40736-x (PMC6416325; doi:10.1038/s41598-019-40736-x)
Supplement: Supplementary file 1 — Supplementary Information [file 41598_2019_40736_MOESM1_ESM.pdf]

Low-dose pioglitazone can ameliorate learning and memory impairment in a mouse model of dementia by increasing LRP1 expression in the hippocampus

Hannah Seok[1,#], Minyoung Lee[2,#], Eugene Shin[3], Mi Ra Yun[4], Yong-ho Lee[2], Jae Hoon Moon[5], Eosu Kim[6], Phil Hyu Lee[7], Byung-Wan Lee[2], Eun Seok Kang[2], Hyun Chul Lee[2], Bong Soo Cha[2,3]*

[1]Department of Internal Medicine, The Catholic University of Korea College of Medicine, Uijeongbu St.Mary's Hospital, Uijeongbu, Korea

[2]Department of Internal Medicine, Yonsei University College of Medicine, Seoul, Korea

[3]Institute of Endocrine Research, Yonsei University College of Medicine, Seoul, South Korea

[4]Brain Korea 21 Project for Medical Science, Yonsei University College of Medicine, Seoul, Korea

[5]Department of Internal Medicine, Seoul National University Bundang Hospital, Seongnam-si, Korea

[6]Department of Psychiatry, Yonsei University College of Medicine, Seoul, Korea

[7]Department of Neurology, Yonsei University College of Medicine, Seoul, Korea

[#]These two authors have contributed equally to this work and are sharing first authorship.

*Corresponding author: Bong Soo Cha

Division of Endocrinology and Metabolism

Department of Internal Medicine, Yonsei University College of Medicine, 50-1, Yonsei-ro, Seodaemun-gu, Seoul 03722, Republic of Korea

Tel: 82-2-2228-2265, Fax: +82-2-393-6884, E-mail: bscha@yuhs.ac

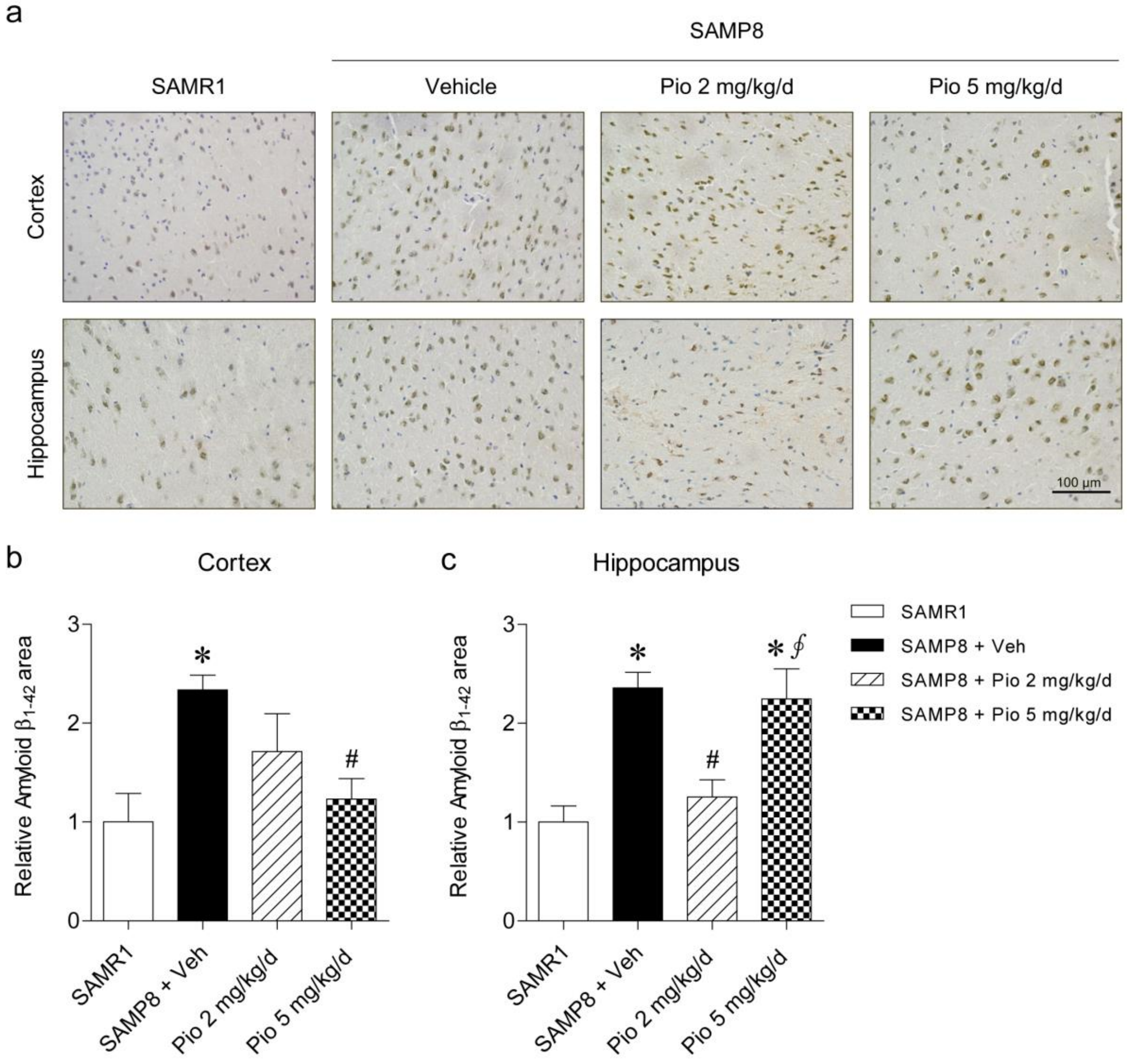

Supplementary Fig. S1 Immunohistochemistry for A$\beta_{1\text{-}42}$ deposits in cortices and hippocampi of SAMR1 and SAMP8 mice. **(a)** Representative immunohistochemical staining images are shown. The relative area covered by A$\beta_{1\text{-}42}$ plaques in **(b)** cortices and **(c)** hippocampi of SAMR1 and SAMP8 mice was analysed. A$\beta_{1\text{-}42}$ deposits were significantly increased in cortices and hippocampi of SAMP8 mice compared to SAMR1 mice. SAMP8 mice treated with 2 mg/kg/day of pioglitazone exhibited significantly reduced A$\beta_{1\text{-}42}$ plaques in the hippocampal area. Values are the mean ± standard error of the mean (n = 8 per group). *$p < 0.05$ compared to SAMR1 mice. #$p < 0.05$ compared to vehicle-treated SAMP8 mice. ∮ $p < 0.05$ compared to SAMP8 mice treated with 2 mg/kg/day of pioglitazone.

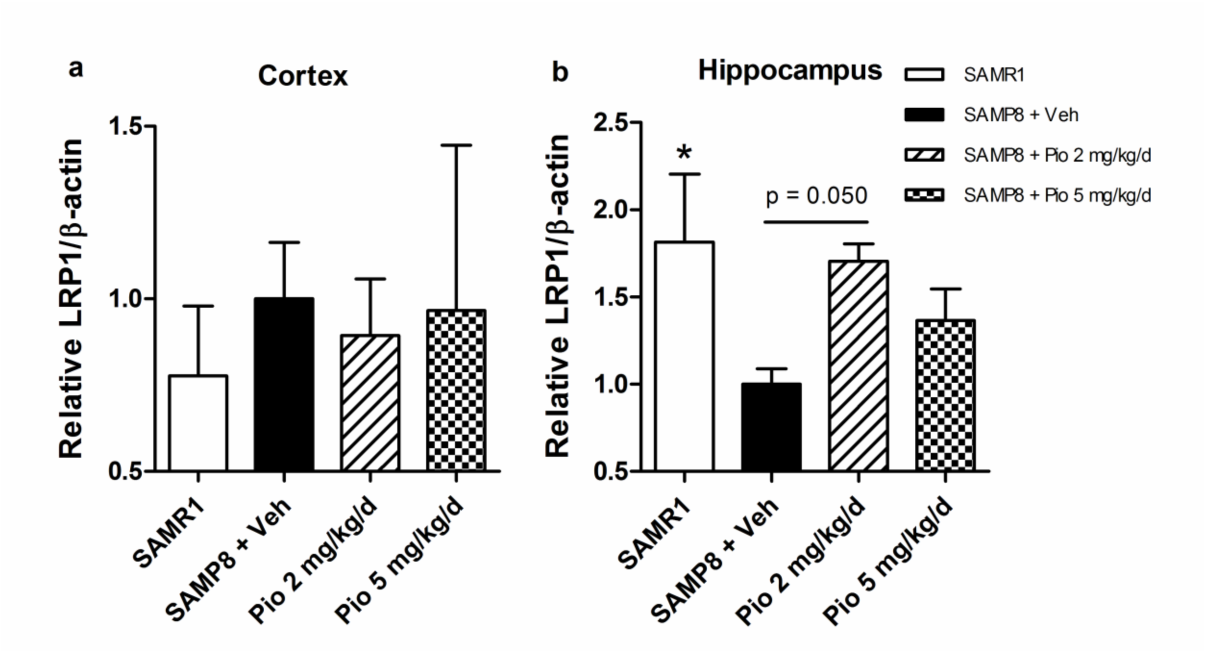

Supplementary Fig. S2 Expression of LRP1 in **(a)** cortices and **(b)** hippocampi of SAMR1 and SAMP8 mice. The relative levels of LRP1 in the cortex and hippocampus were determined by western blot. Vehicle-treated SAMP8 mice were used as the reference group. SAMP8 mice treated with 2 mg/kg/day of pioglitazone exhibited higher levels of hippocampal LRP1 than vehicle-treated SAMP8 mice. Data are the mean ± standard error of the mean (n = 3 per group). *$p < 0.05$ compared to vehicle-treated SAMP8 mice.
